# TNF inhibitors induce discoid fibrosis in the sublining layers of the synovium with degeneration of synoviocytes in rheumatoid arthritis

**DOI:** 10.1007/s00296-013-2743-y

**Published:** 2013-04-11

**Authors:** Shunsei Hirohata, Tetsuya Tomita, Hideki Yoshikawa, Masahisa Kyogoku

**Affiliations:** 1 Department of Rheumatology and Infectious Diseases, Kitasato University School of Medicine, 1-15-1 Kitasato, Minami-ku, Sagamihara, Kanagawa, 252-0374 Japan; 2 Osaka University Medical School, Suita, Osaka, 565-0871 Japan; 3 Department of Pathology, Tohoku University School of Medicine, Sendai, Miyagi, 980-8575 Japan

**Keywords:** Infliximab, Etanercept, Synovium, Histology, Fibrosis, Osteoclast

## Abstract

We determined the characteristic features of synovial tissues of rheumatoid arthritis (RA) patients treated by TNF inhibitors in order to delineate their mechanism of action. Synovial tissues were obtained during the joint surgical operations from 12 RA patients who had been treated with TNF inhibitors in addition to disease modifying antirheumatic drugs (DMARDs) for at least 5 months (5–25 months) (RA-TNFinh), and from 12 RA patients who had been treated with DMARDs alone (RA-DMARD), and were evaluated under light microscopy. There were no significant differences in disease duration, serum CRP levels, DAS28, Steinbrocker’s stages on X-ray and treatment regimen except for TNF inhibitors between RA-TNFinh and RA-DMARD. The most prominent changes in the synovium from RA-TNFinh were discoid fibrosis in the subliming layers of the synovium with degeneration and detachment of synoviocytes and marked decrease in vasculatures. There was no significant difference in these synovial features between RA patients with infliximab and those with etanercept. Interestingly, appearance of osteoclasts was observed in RA-TNFinh (3 out of 12 patients) and in RA-DMARD (1 out of 12 patients). These results indicate that not only infliximab, but etanercept might have direct actions on synovial cells in the deep lining layers of the synovium, leading to the discoid fibrosis thereof. Moreover, the data confirm that the deep lining or sublining layers of the synovium are the most important portions that steer the disease process of RA synovitis.

## Introduction

Rheumatoid arthritis (RA) is a chronic inflammatory disease characterized by hyperplasia of synovial tissues, leading to the destruction of joint structures [1]. Thus, joints in RA consist of massive proliferating synovial tissue, forming invading pannus, resulting in the destruction of cartilage and bone. The characteristic histologic features of the synovium in RA include cellular proliferation in the lining layers as well as in the sublining layers [2]. The lining layer consists mainly of type A and type B synoviocytes, alternatively called intimal macrophages and fibroblast-like synoviocytes, respectively [2–4]. In the sublining layers, there is infiltration of a variety of cells, including dendritis cells, lymphocytes, plasma cells and polymorphonuclear leukocytes [5, 6]. In addition, the formation of pseudo-germinal center, consisting of CD20+ B cells in the center surrounded by CD4+ T cells, is occasionally observed [6]. Another important feature in the synovium of RA is accelerated angiogenesis [7], which presumably results from increased production of angiogenic growth factors by lining cells and inflammatory cells [7]. It should be noted, however, that the synovium of RA also showed the neovascularization in the areas without infiltration of inflammatory cell or proliferation of lining cells, suggesting that angiogenesis might be most proximal to the etiology of RA [8].

TNF inhibitors, including anti-TNF-α antibodies and soluble TNF receptors, have brought epoch-making impacts on treatment for RA [9]. Several studies have proposed the mechanisms of action for TNF inhibitors in RA. Thus, TNF inhibitors have been shown to directly act on inflammatory cells in addition to neutralization of TNF-α [10, 11]. However, the effect of TNF inhibitors on the synovial histology has not been well understood. The current studies were therefore designed to determine the characteristic features of synovial tissues of RA patients treated by TNF inhibitors. To evaluate the specific effects of TNF inhibitors strictly, control RA patients with similar disease activities who had been treated with DMARDs alone were included.

## Patients and methods

### Patients

Synovial tissues were obtained from 12 RA patients who had been treated with TNF inhibitors (3 patients with infliximab, 9 patients with etanercept) for at least 5 months (5–25 months) during the joint surgical operations. As controls, synovial tissues were similarly obtained from 12 RA patients who had not received TNF inhibitors. Most of the operations were total joint replacement orthopedic surgery of hip, knee or elbow joints, as shown in Table 1. All the patients satisfied the American College of Rheumatology 1987 revised criteria for RA [12] and gave informed consent in accordance with the World Medical Association Declaration of Helsinki Ethical Principles for Medical Research Involving Human Subjects. The demographic features and treatment regimen in the 24 patients are shown in Table 1. This study was conducted with the approval of the ethical committee of Osaka University Medical School.

**Table 1:** Profile of the patients

Control									With TNF inhibitors								
Age	Gender	Disease duration	Class	Treatment	Ope	CRP	DAS28	X-p stage	Age	Gender	Disease duration	Class	Treatment	Ope	CRP	DAS	X-p stage
72	F	25	4	Tac	TAA	3.00	5.33	4	49	M	21	4	Eta, Tac, PSL	THA	4.40	4.92	3
67	F	17	4	MTX	THA	1.10	4.04	3	52	F	28	3	Eta, MTX	TKA	1.70	5.11	4
43	F	22	4	PSL	THA	1.24	4.56	4	41	F	19	3	Eta, MTX	TKA	0.30	4.13	4
60	F	17	3	(–)	TKA	0.46	3.59	3	45	F	12	3	Eta, Tac, PSL, MTX	TEA	0.57	3.75	3
59	M	10	3	PSL, MTX	TKA	1.41	4.54	4	50	M	7	4	Inf, Tac, PSL, MTX	THA	0.17	2.95	3
51	F	18	2	PSL, Tac	FoAp	3.90	5.23	3	66	F	22	3	Eta, PSL	TKA	0.44	3.67	4
59	F	19	4	PSL, MTX, Tac, BUC	TKA TEA	0.34	3.87	4	33	F	8	2	Eta, PSL, MTX	WAp	0.67	4.4	3
55	F	38	3	Tac, MTX	TKA	0.31	3.21	4	30	F	18	2	Eta, PSL, MTX, Tac	ESy	1.23	3.86	4
71	F	18	4	PSL, MTX	TKA	0.30	3.69	3	56	F	19	3	Inf, PSL, MTX	TEA	2.95	4.88	4
62	F	38	2	PSL, SASP	FiAp	0.04	2.97	4	62	F	23	3	Inf, PSL, Tac, MTX	TKA	7.16	6.22	3
38	F	17	3	PSL, SASP, MTX	TKA	0.80	3.78	3	51	F	10	4	Eta, PSL, SASP, MTX	THA	0.63	4.13	3
63	F	21	3	SASP	TKA	1.80	3.62	3	53	F	13	2	Eta, PSL, BUC, MTX	FoAp	0.68	4.78	4
Mean		21.7				**1.23**	**4.04**		Mean		16.7				**1.74**	**4.40**	

*PSL* prednisolone, *MTX* Mthotrexate, *BUC* Bucillamine, *Tac* Tacrolimus, *SASP* Sulfasalazopyridine, *Eta* etanercept, *Inf* infliximab, *TAA* total ankle arthroplasty, *THA* total hip arthroplasty, *TKA* total knee arthroplasty, *TEA* total elbow arthroplasty, *FoAp* foot athroplasty, *FiAp* finger arthroplasty, *WAp* wrist arthroplasty, *ESy* elbow synovectomy

### Synovial tissue histology

Synovial tissues were fixed in formaldehyde and embedded in paraffin. The sections were stained by hematoxylin and eosin, Masson’s trichrome, immunohistochemistry with anti-CD68 monoclonal antibody (clone KP-1) and tartrate-resistant alkaline phosphatase (TRAP), followed by evaluation under the light microscopy.

## Results

### Patients’ backgrounds

As shown in Table 1, there were no significant differences in disease duration [21.7 ± 8.4 years (mean ± SD) vs. 16.7 ± 6.6 years], serum CRP levels (1.74 ± 2.10 mg/dl vs. 1.23 ± 1.18 mg/dl), DAS28 (DAS28-CRP with 3 variables) (4.04 ± 0.74 vs. 4.40 ± 0.85), Steinbrocker’s stages on X-ray and Steinbrocker’s functional classes between RA patients with TNF blockers and those without TNF inhibitors. As for treatment, both groups have similar treatment regimen except for the use of TNF inhibitors. Thus, 7 and 10 patients were with prednisolone, 6 and 10 patients were with methotrexate and 3 and 4 patients were with tacrolimus in the control group and in the TNF inhibitors group, respectively.

### Histological features of the synovium

**Fig. 1:**
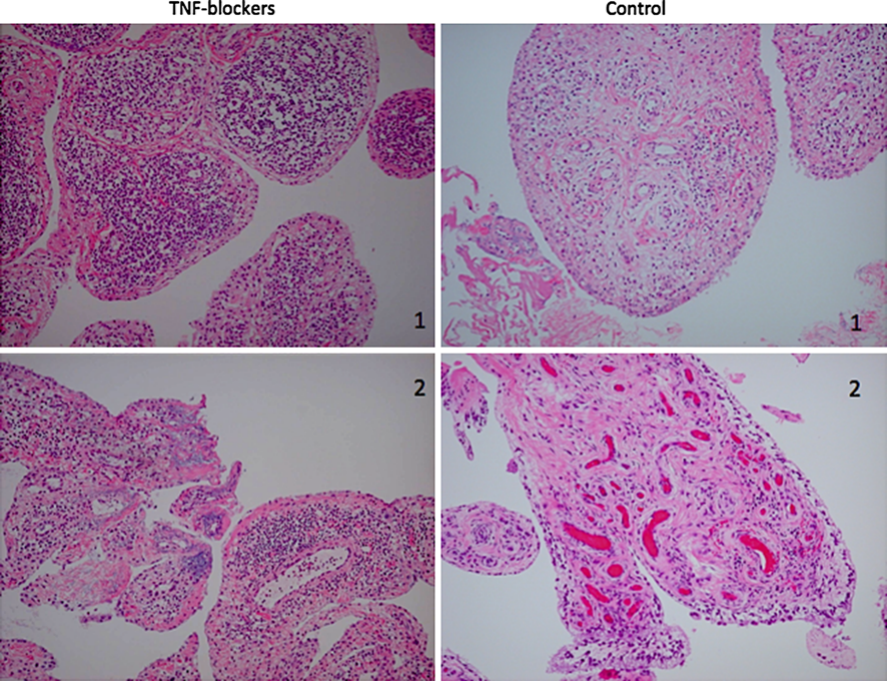
Comparative histological changes in the synovium of RA patient treated with TNF blockers and control cases. *Left* TNF blockers: thinning of synovial lining layer (*1*) and discoid fibrosis of lining and sublining zone (*2*), *Right* control cases: diffuse edematous villi (*1*) and diffuse fibrosis with many dilated vasculature (*2*) in the control cases. Hematoxylin and eosin, original magnification ×40

**Fig. 2:**
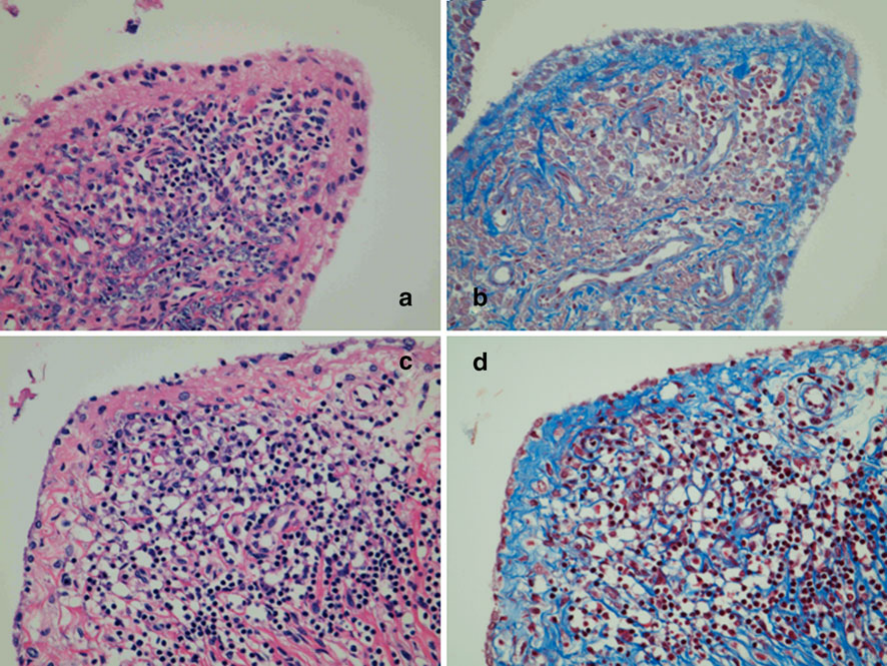
Discoid fibrosis in the synovium of RA patient treated with TNF blockers. *Left* hematoxylin and eosin, original magnification ×100. *Right* Masson’s trichrome, original magnification ×100. **a**, **b** The same synovial tissue of TNF blockers (*1*) in Fig. 1. **c**, **d** The same synovial tissue of TNF blockers (*2*) in Fig. 1

The most prominent change in the synovium from RA patients with TNF inhibitors (infliximab and etanercept) was discoid fibrosis in the sublining layers, which was almost absent in the synovium of control patients without TNF inhibitors (Fig. 1). By contrast, though diffuse edematous villi and diffuse fibrosis with many dilated vasculatures were observed, we could not find any typical discoid fibrosis in the sublining layers in the control synovial tissues. The formation of discoid fibrosis in patients with TNF inhibitors was further confirmed in sections with Masson’s trichrome stain (Fig. 2).

**Fig. 3:**
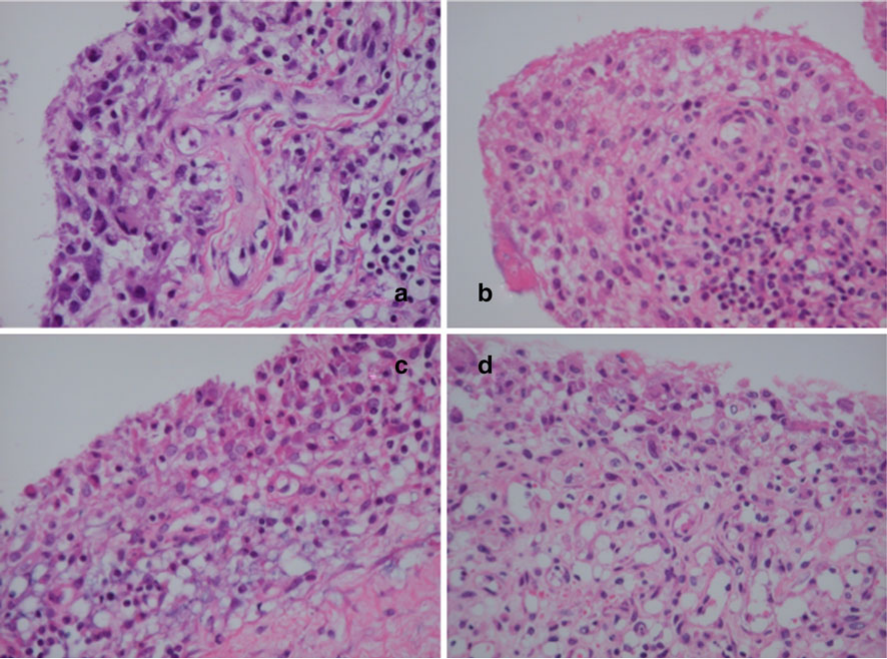
Typical histological changes in the synovium of the RA patients treated with TNF blockers (*1*). **a** Pyknosis of surface synovial cells and sclerosis of small vasculature in the subsynovial space are clear. **b** Hydropic degeneration of surface synovial cells. **c** Vacuolation of deeper synovial cells are evident. **d** Same as **c** and more evident. Hematoxylin and eosin, original magnification ×100

**Fig. 4:**
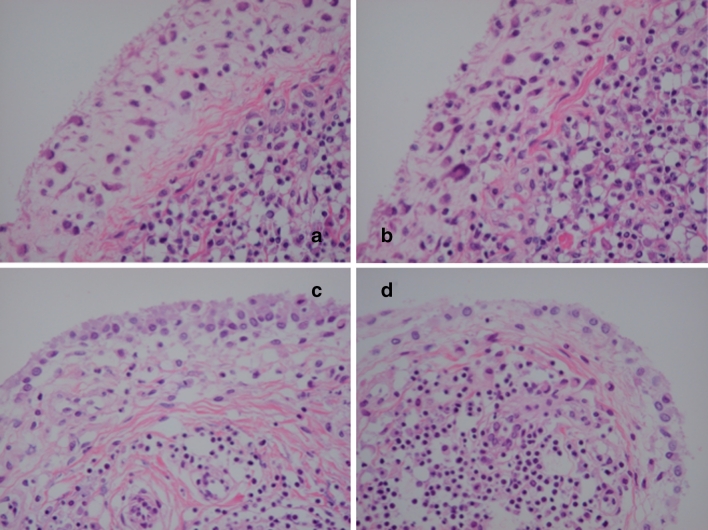
Typical histological changes in the synovium of the RA patients treated with TNF blockers (*2*). **a** Almost complete hydropic degeneration of synovial cells with discoid (board-like) fibrosis. **b** Same as **a** with some synovial cell derived giant cells. **c** Just one or two layers of synovial membrane are *left* and sublining fibrosis with sclerosis of small vasculature are starting. **d** Same as **c** with more lymphocytes *left*. Hematoxylin and eosin, original magnification ×100

**Fig. 5:**
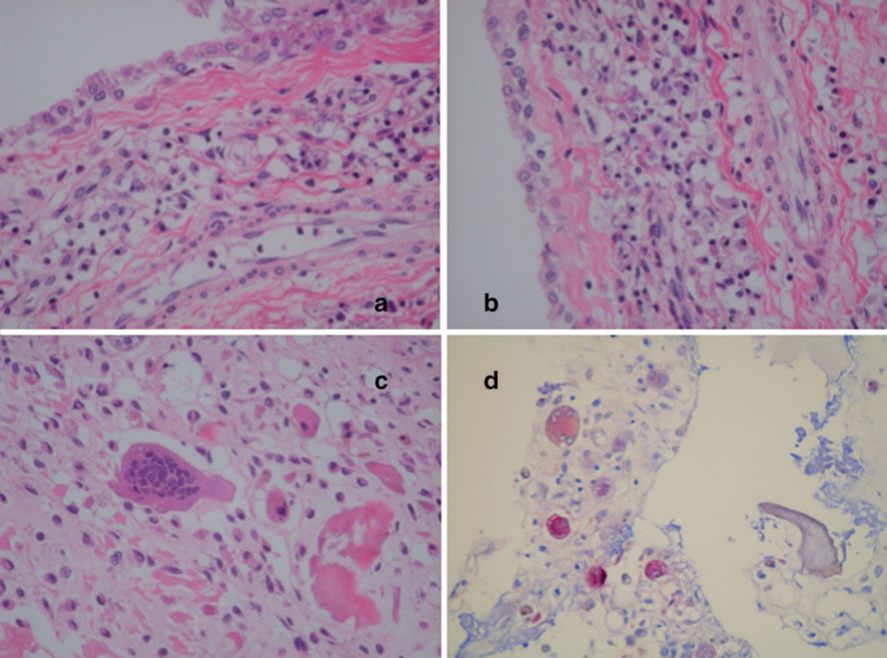
Typical histological changes in the synovium of the RA patients treated with TNF blockers and additional peculiar histological changes: TRAP-positive osteoclasts. **a** Discoid fibrosis in the subsynovial area is evident. The characteristic feature of RA synovium treated with TNF blocker. **b** Same as **a**. Perivascular fibrosis in the sublining area is also clear. Hematoxylin and eosin, original magnification ×100. The giant cells are located around the debris of synovium-attacked and destructed bone and/or chondroid matrix. They must be a symbol of acceleration of clearing and healing. Hematoxylin and eosin (**c**), and TRAP staining (**d**). Original magnification ×100 (**c**), ×40 (**d**)

In some portions, several characteristic changes appear to precede the formation of the discoid fibrosis in the synovium of patients with TNF inhibitors, including (1) pyknosis of surface synovial cells, (2) hydropic degeneration of surface synovial cells, (3) vacuolization in the deep lining layers and (4) sclerosis of small vasculature in the subsynovial space (Fig. 3). In other portions, almost complete hydropic degeneration of synovial cells with discoid (board-like) fibrosis was noted. There were some giant cells in the lining layers. It appears that after these degeneration events, just one or two layers of synovial membrane were left with sublining fibrosis with sclerosis of small vasculature (Fig. 4), leading to the appearance of marked discoid fibrosis and perivascular fibrosis in the sublining area with the lining layer consisting of only two layers of synoviocytes (Fig. 5).

**Fig. 6:**
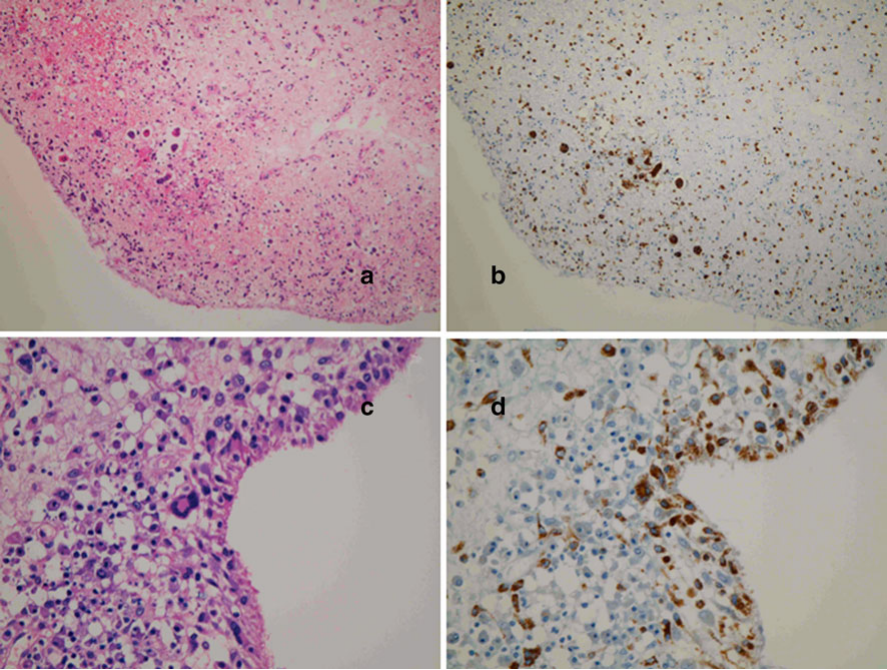
Formation of giant cells in the lining and sublining layers in the synovium of RA patient treated with TNF blockers. *Left* hematoxylin and eosin, original magnification ×40 (**a**), ×100 (**c**). *Right* immunohistochemistry with anti-CD68 monoclonal antibody (clone KP-1), original magnification ×40 (**b**), ×100 (**d**). **a**, **c** Several giant cells in the sublining layer. **b**, **d** Several giant cells in the lining and sublining layers

Interestingly, another type of polynuclear giant cells which are TRAP positive, indicating that they are osteoclasts, were observed in RA patients with TNF inhibitors (3 patients with etanercept out of 12 patients) and in those without TNF inhibitors (1 out of 12 patients). Notably, appearance of these giant cells is usually accompanied by cartilage debris clearance and bone formation (Fig. 5d). The giant cells in the sublining layers as well as those in the lining layers were positive for CD68 (Fig. 6), indicating that they were derived from type A synoviocytes.

Table 2 summarizes the histopathological features of the synovial tissues from RA patients with or without TNF inhibitors. As for changes in surface synovial cells, hydropic degeneration and vacuolation were observed more frequently in patients with TNF inhibitors than in those without TNF inhibitors. Degeneration of synoviocytes in the deep lining layers as well as formation of discoid fibrosis in the sublining layers were observed in nearly all the patients with TNF inhibitors. In addition, narrowing or obstruction of vasculatures in the sublining layers was more frequently observed in patients with TNF inhibitors, although it did not reach the statistical significance. These results indicate that not only infliximab, but etanercept might have direct actions on synovial cells, presumably synoviocytes in the deep lining layers of the synovium, leading to the discoid fibrosis in the sublining layers. Moreover, the data confirm that the sublining layers of the synovium are the most important portions that steer the disease process of RA synovitis.

**Table 2:** Effects of TNF inhibitors on the synovial histopathology in RA

	Control group *n* = 12	TNF inhibitors *n* = 12
Changes in surface synovial cells		
Pyknosis	9	6
Hydropic degeneration	5	11*
Vacuolation	4	11*
Degeneration in the sublining layers, including formation of giant cells	4	12*
Discoid fibrosis in the sublining layers	2	11*
Loss, narrowing or obstruction of vasculature in the sublining layer	2	7
Congestion or dilatation of vasculature in the sublining layer	7	2
Extensive scarring	3	2
Osteoclast	1	3

* *P* < 0.05 by Fisher’s exact test

## Discussion

The current studies have disclosed the characteristic features of synovial tissues of RA patients who had been treated with TNF inhibitors in comparison with those of RA patients with DMARDs alone. There were no significant differences in disease duration, serum CRP levels, DAS28 (DAS28-CRP with 3 variables), Steinbrocker’s stages on X-ray and Steinbrocker’s functional classes between both groups. It should be pointed out that all the patients in both groups had significant structure damages that required surgery. Therefore, it is most likely that both groups presented comparable RA disease activities. Moreover, there were no significant differences in treatment regimen between both groups, except for the use of TNF inhibitors. There is no difference between etanercept and infliximab, but similar changes could not be observed in patients given tocilizumab. Thus, discoid fibrosis in the sublining layers was observed in none of the 5 patients with tocilizumab (data not shown). Therefore, it is most likely that the histopathological features in the synovium of the RA patients with TNF inhibitors might be caused by TNF inhibitors themselves. Nonetheless, further studies with a larger number of patients with tocilizumab or abatacept would be important for the confirmation of the characteristic histopathological features in the synovium of the RA patients with TNF inhibitors. It would be also intriguing to compare the patients with TNF inhibitor alone and those with TNF inhibitors and MTX, since it is now well known that TNF inhibitor monotherapy is not enough to prevent joint destruction [11].

The most prominent features of synovial tissues in RA patients treated with TNF inhibitors were discoid fibrosis in the sublining layers with various types of degeneration and detachment of synoviocytes in the deep lining layer, which could be observed in all the patients treated with TNF inhibitors. Discoid fibrosis appeared to be associated with vacuolation of degenerating synovial cells. There was no significant difference in the synovial histological features between RA patients with infliximab and those with etanercept. It is suggested that degenerating synoviocytes might be type B synoviocytes, which have been demonstrated to be located in the deep lining layers [2]. Notably, the outside-in signals delivered by infliximab, but not etanercept, through membrane bound TNF-α result in apoptosis of TNF-α-expressing Jurkat cells [14]. However, it has been shown that infliximab as well as etanercept induced apoptosis in human peripheral blood monocytes [15]. Since type B synoviocytes have also been shown to express TNF-α like monocytes on their surface [13], it is possible that infliximab and etanercept might bind and deliver signals leading to degeneration or apoptosis. On the other hand, recent studies have shown that differentiation of type B synoviocytes requires the action of TNF-α [16, 17]. Therefore, it is also possible that inhibition of TNF-α might suppress the differentiation of type B synoviocytes within the synovium, leading to the loss or decreased stratification of synovial lining cells.

The other characteristic feature is the loss, narrowing or obstruction and fibrosis of vasculatures in the sublining layers, which could not be observed in the synovial tissues of RA patients treated with DMARDs alone. There, the preservation of vasculatures was clearly observed in the synovial tissues, but around them, synovial cell proliferation and inflammatory cell infiltration were markedly decreased or even disappeared and even covered with diffuse fibrosis. Therefore, the loss or obstruction of vasculatures due to TNF inhibitors could not be accounted for by decreased production of angiogenic cytokines, such as vascular endothelial growth factor (VEGF). In this regard, we previously demonstrated that angiogenesis took place prior to the proliferation of synovial lining cells or infiltration of inflammatory cells in the synovial tissues of very early stages of RA [8]. The results in the current studies further support the hypothesis that angiogenesis might not be secondary to inflammatory cell infiltration, but rather a primary event in the RA synovium. Thus, it is likely that the loss of vasculatures caused by TNF inhibitors might lead to the degeneration and detachment of synoviocytes as well as the formation of discoid fibrosis in the sublining layers.

We have previously disclosed that abnormalities in bone marrow (BM) CD34+ cells are involved in the angiogenesis in the RA synovium. Thus, RA BM CD34+ cells have enhanced capacities to differentiate into endothelial cells in relation to synovial vascularization [18]. The enhanced expression of VEGF receptor 2/KDR mRNA in RA BM CD34+ cells might account for their enhanced capacities to differentiate into endothelial cells [18]. On the other hand, previous studies disclosed that TNF-α up-regulates the expression of VEGF receptor 2 in human vascular endothelial cells [19] and thus promotes angiogenesis [20]. In fact, recent studies have also revealed that immature blood vessels in RA synovium are selectively depleted in response to anti-TNF-α treatment [21]. Thus, most RA synovial tissues contained a significantly greater fraction of immature blood vessels lacking periendothelial coverage, which were observed from the earliest phases of the disease and were depleted by anti-TNF-α therapy [21]. Taken together, it is most likely that the formation of immature blood vessels might be the most crucial process in the pathogenesis of RA, which is inhibited by anti-TNF-α treatment.

In some of the RA synovial tissues, the formation of multinucleated giant cells was observed, especially in patients treated with TNF inhibitors. Some of the giant cells appeared in the synovial lining layers to be a result of degenerative process, but others with multiple nuclei were TRAP-positive, indicating that they are osteoclasts. The mechanism of the formation of osteoclasts especially in the synovium of patients with TNF inhibitors remains uncertain. It should be noted that infliximab, but neither anti-TNF receptor p55 antibody nor anti-TNF receptor p75 antibody, enhances osteoclast formation from human osteoclast progenitors in vitro [22]. Therefore, the formation of osteoclast in the synovium of patients with TNF inhibitors is considered to result from their direst actions on osteoclast progenitors, such as monocyte lineage cells. Although the formation of osteoclast-like cells was accompanied by the bone formation, the precise mechanism for their appearance remains currently unclear. Further studies are required to clarify the mechanism and significance of osteoclast formation in the synovium.

In summary, the current studies have disclosed that TNF inhibitors result in the degeneration and disappearance of synovial cells in the deep lining layers, leading to the discoid fibrosis of characteristic morphology in the sublining layers. Moreover, the loss, narrowing or obstruction of vasculatures in the sublining layers is another characteristic feature of the synovium in RA patients treated with TNF inhibitors. These data confirm that the deep lining or sublining layers of the synovium are the most important portions that steer the disease process of RA. To elucidate the role of TNF-α in detail especially in these regions would be helpful for the complete understanding of the pathogenesis of RA.
